# *TaMAPK4* Acts as a Positive Regulator in Defense of Wheat Stripe-Rust Infection

**DOI:** 10.3389/fpls.2018.00152

**Published:** 2018-02-15

**Authors:** Bing Wang, Na Song, Qiong Zhang, Ning Wang, Zhensheng Kang

**Affiliations:** ^1^State Key Laboratory of Crop Stress Biology for Arid Areas and College of Life Sciences, Northwest A&F University, Yangling, China; ^2^State Key Laboratory of Crop Stress Biology for Arid Areas and College of Plant Protection, Northwest A&F University, Yangling, China

**Keywords:** host defense mechanisms, mitogen-activated protein kinase, *Puccinia striiformis*, wheat, virus-induced gene silencing

## Abstract

Highly conserved mitogen-activated protein kinase (MAPK) cascades regulate numerous plant processes, including hormonal responses, stress, and innate immunity. In this research, *TaMAPK4* was predicted to be a target of tae-miR164. We verified the binding and suppression of *TaMAPK4* by co-expression in *Nicotiana benthamiana*. Moreover, we found *TaMAPK4* was localized in the cytoplasm and nucleus using transient expression analyses. *TaMAPK4* transcripts increased following salicylic acid (SA) treatment and when host plants were infected with an avirulent race of the stripe-rust pathogen. Silencing of *TaMAPK4* by virus-induced gene silencing permitted increased colonization by the avirulent pathogen race. Detailed histological results showed increased *Puccinia striiformis* (*Pst*) hyphal length, hyphal branches, and infection uredinial size compared to the non-silenced control. SA accumulation and the transcript levels of *TaPR1, TaPR2*, and *TaPR5* were significantly down-regulated in *TaMAPK4* knockdown plants. Overall, these results suggest that *TaMAPK4* plays an important role in signaling during the wheat-*Pst* interaction. These results present new insights into MAPK signaling in wheat defense to rust pathogen.

## Introduction

MicroRNAs (miRNAs) are negative post-transcriptional regulators of gene expression and play important roles in multiple biological processes, including plant immunity (Bartel, 2004; Navarro et al., 2006). The conserved plant miRNA miR164 regulates multiple target genes through cleavage to mRNA (Kim et al., 2009). miR164 participates in a range of physiological processes, including flower development, age-dependent cell death, and plant defense (Laufs et al., 2004; Kim et al., 2009; Li et al., 2014). Plants miR164 plays important roles in responding to biotic stresses; this molecule regulates the transcript levels of the genes to which it binds. For example, miR164 was induced in rice inoculated with *Magnaporthe oryzae* (Li et al., 2014). *TaNAC21* in wheat is a target gene of miR164, leading to increase susceptibility to stripe-rust in wheat (Feng et al., 2014). In previous research, we predicted that miR164 binds to a mitogen-activated protein kinase (MAPK) gene in wheat (Wang et al., 2014). MAPK cascades are pivotal signal transduction modules in plants.

Mitogen-activated protein kinase pathways are evolutionarily conserved across the animal and plant kingdoms (Tena et al., 2001). Three kinase classes, including MAPKKK, MAPKK, and MAPK, compose a typical MPK cascade (Hao et al., 2015). Multiple MAPK genes have been described in plants, including Arabidopsis, tomato, and rice (Zhang and Klessig, 1998; Cardinale et al., 2002; Hamel et al., 2006).

Mitogen-activated protein kinase cascades play important roles in plant defense involving pathogen-associated molecular pattern (PAMPs)-triggered immunity (PTI), which was composed of plant-innate immunity (Jones and Dangl, 2006; Cristina et al., 2010). The best characterized MAPKs are MPK3 and MPK6 in Arabidopsis, which play positive roles in regulating of the defense response (Pitzschke et al., 2009). Pathogen effectors suppress MAPK activation to override plant defense responses. For example, MPK3 and MPK6 are inactivated by bacterial pathogen effector HopAI1 to suppress PAMP-induced genes (Hématy et al., 2009). Moreover, MAPK also can play negative regulatory roles in plant defense (Petersen et al., 2000). In tobacco (*Nicotiana tabacum*), the genes controlling salicylic acid-induced protein kinase (SIPK) are activated by various pathogen-related signals (Zhang and Klessig, 1998). An MAPK gene in rice, BWMK1, mediates the expression of many pathogenesis-related genes by the activation of the transcription factor OsEREBP1 (rice ethylene-responsive element-binding protein 1), resulting in increased resistance to pathogens (Cheong et al., 2003). MAPK cascades participate in various plant defense signaling processes such as biosynthesis and the subsequent signaling of salicylic acid (SA) (Petersen et al., 2000; Beckers et al., 2009). In the present study, we predicted one candidate target of miR164 in wheat was *TaMAPK4* (not the homolog of Arabidopsis *MPK4*), which involved wheat responses to multiple stresses (Hao et al., 2015). *TaMAPK4* shared high-sequence similarities to Arabidopsis MPK1, whose role in the plant defense reaction was still unknown.

Wheat stripe rust, which is caused by *Puccinia striiformis* f. sp. *tritici* (*Pst*), is among the most destructive wheat diseases worldwide, and wheat yield can be greatly reduced or even completely destroyed at an epidemic level (Wang et al., 2017). In the present study, we demonstrate *TaMAPK4* could be regulated by miR164. *TaMAPK4* localizes to the cytoplasm and the nucleus. Moreover, functional characterization supported that *TaMAPK4* is a positive regulator of resistance to *Pst* in wheat. Our results suggest that miRNAs and MAPK signaling might be involved in regulating plant immunity and defense.

## Materials and Methods

### Plant Materials and Inoculation

Su11 is a Chinese wheat cultivar susceptible to *Pst* race CYR31 (compatible interaction) and resistant to CYR23 (incompatible interaction). The method of wheat seedling culture, inoculation, and incubation followed was previously described by Kang et al. (2002). Parallel mock inoculations were performed using tap water. After inoculation for 24 h, the seedlings were transferred to a 14°C growth chamber with a 16-h photoperiod. The control and inoculated wheat leaves were harvested at 0, 12, 24, 48, 72, and 120 h post-inoculation (hpi) and immediately frozen in liquid nitrogen. *Nicotiana benthamiana* was cultured in a growth chamber with a 16-h/8-h photoperiod at 25°C.

### Hormone Treatments

For the hormone treatments, 2-week-old wheat seedlings at the second to third leaf-growth stages were separately sprayed with 2 mM SA, 100 μM methyl jasmonate (MeJA), a 100 μM ethephon (ET) solution, all of which were dissolved in 0.1% (v/v) ethanol (Wang et al., 2017). The control plants were sprayed with 0.1% (v/v) ethanol as for hormone treatments. Leaf samples were harvested at 0, 6, 12, and 24 hours post-treatment (hpt).

### RNA Extraction and Quantitative Real-Time PCR Analysis

A PureLink RNA Mini Kit (Invitrogen, Beijing, China) was used to total RNA extract. A Revert Aid First-strand cDNA Synthesis Kit from Fermentas (Waltham, MA, United States) was used to synthesize cDNA from RNA. Quantitative real-time PCR (qRT-PCR) was performed on a CFX96 Real-Time System (Bio-Rad, Munich, Germany) using SYBR Green I (Invitrogen) for fluorescence. The total volume for PCR was 25 μl. The following PCR conditions were used: 95°C for 1 min, followed by 39 cycles at 95°C for 10 s, 55°C for 30 s, and 72°C for 1 min, with a final cycle at 72°C for 5 min. To standardize the data, the wheat-elongation factor *Ta*EF-1α was used as an endogenous control for qRT-PCR analyses in wheat. The primers used in qRT-PCR are listed in **Supplementary Table S2**. Relative gene expression data were quantified using the comparative 2^-ΔΔC_T_^ method (Livak and Schmittgen, 2001).

### Plasmid Constructions

The primers used for plasmid construction in the present study are listed in **Supplementary Table S2**. *TaMAPK4* was cloned from the cDNA of the wheat cultivar Su11 using FastPfu DNA Polymerase (TransGen Biotech, Beijing). We used *TaMAPK4* in the vector pCAMBIA-1302 as a template to amplify the gene using specific primers with *Pst*I and *Xba*I restriction enzyme sites.

The *tae*-miR164 precursor miR164 was digested using restriction endonucleases *Sac*I and *Bam*HI, and cloned into the pBI121 vector. The region containing the cleavage site in *TaMAPK4* sequences was digested using the restriction endonucleases *Xba*I and *Bam*HI and cloned into the pBI121 vector.

The plasmids used for virus-induced gene silencing (VIGS) were based on previously described constructs (Holzberg et al., 2002). RNA-derived clones (BSMV-*TaMAPK4*) were created using BSMV-*TaPDS* in which wheat phytoene desaturase (*TaPDS*) sequence fragments were replaced with specific *TaMAPK4* sequences. The amplicon of *TaMAPK4* was cut using *Not*I and *Pac*I, and ligated into the sites of the BSMV: γ vector.

### Subcellular Localization

A *TaMAPK4*-GFP fusion vector was introduced into *Agrobacterium tumefaciens* strain GV3101 by electroporation. Strains carrying the different recombinant vectors [empty vector (EV) and pCAMBIA-1302-*TaMAPK4*] were cultured to an optical density of 0.8 at 600 nm (OD_600_) for injection into the leaves of *N. benthamiana* (4-week-old). Infiltrated seedlings were cultured in a growth chamber with a 16-h/8-h photoperiod at 25°C. Infiltrated leaf tissue samples were harvested at 2 days post-infiltration. DAPI was added to the cell suspensions at a concentration of 5 μg ml^-1^ to counter-stain the nuclei. GFP signals were detected using an Olympus BX-51 microscope (Olympus, Corp., Tokyo, Japan).

Next, 200 mg of *N. benthamiana* leaves transformed with *A. tumefaciens* strain containing pCAMBIA-1302-TaMAPK4-eGFP and pCAMBIA-TaMAPK4-eGFP were ground in liquid nitrogen and resuspended in extraction buffer (1 mM EDTA, 50 mM Tris pH 7.4, 1% sodium deoxycholate, 1% SDS, 150 mM NaCl, 5 mM NaF, 1% Triton X-100, 1 mM DTT, and 1 mM PMSF). The proteins were separated on 12% SDS-PAGE gels and transferred onto nitrocellulose blotting membranes (0.45 μm pore size; Bio-Rad, United States) by wet electroblotting. After blocking with 5% milk at room temperature for 2 h, the membranes were incubated with primary antibody (mouse anti-eGFP antibody, Sigma–Aldrich Chemie GmbH, Taufkirchen, Germany) at a 1:1,000 dilution to detect the eGFP-tagged fusion protein. An anti-mouse antibody was used as a secondary antibody at a 1:5,000 dilution (Sigma–Aldrich Chemie GmbH, Taufkirchen, Germany). Protein expression was detected using an enhanced chemiluminescence (ECL) kit (Thermo Fisher Scientific, Waltham, MA, United States).

### Prediction of miR164 RNA Target and Co-transformation of miR164 and *TaMAPK4*

The target gene of miR164 was predicted using the psRNATarget^1^ program at default settings. A maximum of two continuous mismatches were allowed, using a score cutoff ≤3.0.

Co-transformation was based on Feng et al. (2014). *TaMAPK4* and miR164 were each integrated into the pBI121 vector containing the GUS reporter gene. *A*. *tumefaciens* strain GV3101 was used to introduce the recombinant vectors into the same *N. benthamiana* leaves. Strains carrying different recombinant vectors (EV, *TaMAPK4*, and miR164) were cultured to an optical density of 0.8 at OD_600_ prior to injection. The samples were diluted to OD_600_ = 0.5 using 10 mM of MgCl_2_. miR164+*TaMAPK4* were mixed in equal volumes, and the mixture (OD_600_ = 1.0), was used to test for the cleavage activity of miR164. Similarly, the mix of *TaMAPK4* and miR159 was used as a control. Liquid (1 mL) from each treatment was infiltrated into *N. benthamiana* leaves. Histochemical staining and quantification of GUS were performed as described (Jefferson et al., 1987).

### BSMV-Mediated *TaMAPK4* Gene Silencing

Wheat seedlings were infected with BSMV (barley-stripe mosaic virus) using the method described by Hein et al. (2005). The wild-type seedlings were used as a control check (CK). *TaPDS* (phytoene desaturase) was used as a positive control. The wheat seedlings were inoculated with each of the three viruses (BSMV: γ, BSMV: *TaPDS*, and BSMV: *TaMAPK4*). Ten days after virus inoculation, the fourth leaves were infected with urediniospores of races CYR23 (avirulent) or CYR31 (virulent). The fourth leaves were sampled at 0, 24, 48, and 120 hpi with *Pst* for cytological observation and RNA isolation.

The silencing efficiency of *TaMAPK4* was calculated from the qRT-PCR data. To estimate changes in the fungal biomass, the single-copy target genes *PstRTP1* (rust transferred protein 1 of *Pst*) and *TaEF1* were used to analyze the slopes of the standard curves (Panwar et al., 2013).

For SA analysis, 200 mg of fresh leaf tissue per sample was ground in liquid nitrogen and used to extract SA for HPLC-MS (API 2000; AB SCIEX, Framingham, MA, United States) as previously described (Segarra et al., 2006).

### Histological Determination of Fungal Growth

The harvested samples were decolorized as previously described (Wang et al., 2007). The staining procedure for wheat germ agglutinin (WGA) conjugated to the fluorophore Alexa 488 (Invitrogen, Carlsbad, CA, United States) was used to obtain high-quality images of the developing uredinia. The cleared wheat leaf segments were examined using an Olympus BX-51 microscope (Olympus, Corp., Tokyo, Japan). Five randomly selected leaf segments were examined to record fungal development and defense responses in infected host cells. The hyphal lengths and infection areas were measured using DP-BSW software (Olympus, Corp., Tokyo, Japan). One-way analysis of variance was performed using SPSS (IBM^2^). Three independent biological replicates were performed.

## Results

### Tae-miR164 Regulates Expression of *TaMAPK4*

**FIGURE 1:**
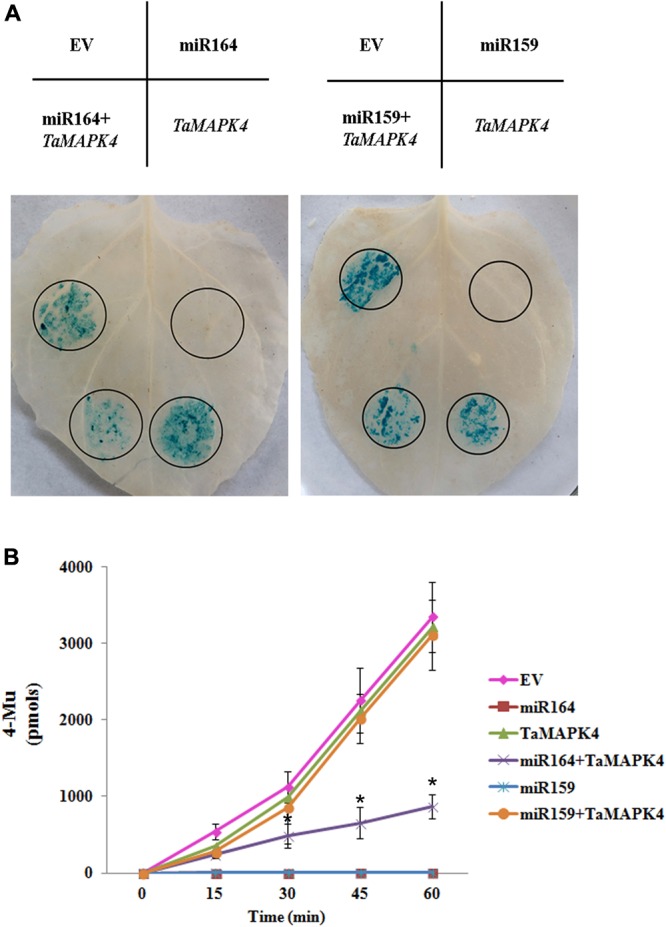
Co-expression of tae-miR164 and *TaMAPK4* in *Nicotiana benthamiana* leaves. **(A)** β-glucuronidase (GUS) phenotypes observed by histochemical staining. **(B)** Quantitative detection of GUS activity in leaves inoculated with different recombinant vectors at different time points using a fluorospectrophotometer. EV, empty vector; 4-MU, 4-methyl-umbelliferyl- β-D-glucuronide. The results are presented as the means ± standard errors of three biological replications. Significant differences were determined using Student’s *t*-test: ^∗^*P* < 0.05.

In this study, we predicted target genes of miR164 in wheat, and found that one candidate target of miR164 in wheat is *TaMAPK4* (**Supplementary Table S1**). To verify the suppression of *TaMAPK4* by miR164, we co-expressed factors in *N. benthamiana* (**Figure 1A**). A recombinant pBI121 vector containing the β-glucuronidase (GUS) as a reporter was introduced into *N. benthamiana* leaves using the *Agrobacterium*-mediated transformation system. The pBI121 EV was used as a control. Leaves infiltrated with pBI121-*TaMAPK4*, in which the target sequence was fused upstream of the GUS gene, showed a similar phenotype to the EV. The pBI121-pre-miR164 construct produced no GUS phenotype in the leaf, in which the GUS gene was replaced by the precursor of miR164. However, GUS staining was markedly reduced in leaves co-expressing the mixture (pBI121-*TaMAPK4* and pBI121-pre-miR164). Moreover, we used miR159 as a control, which shared no sequence similarity with miR164. Co-expression of the mixture (pBI121-*TaMAPK4* and pBI121-pre-miR159) showed a similar phenotype to the EV.

To confirm the histochemical results, the levels of GUS in the leaves were measured using a fluorospectrophotometer (**Figure 1B**). Fluorescence in the EV, pBI121-*TaMAPK4* and the mixture treatments (pBI121-*TaMAPK4* and pBI121-miR159) in the inoculated leaf samples increased with reaction times. These results indicated that GUS was still present and functional. No fluorescence was detected in leaves injected with pBI121-miR164 or pBI121-miR159. Compared with the EV treatments, fluorescence with the mixture (pBI121-*TaMAPK4* and pBI121-miR164) showed a slow increase, demonstrating that miR164 effectively regulated *TaMAPK4*.

### TaMAPK4 Protein Is Localized in the Nucleus and Cytoplasm

**FIGURE 2:**
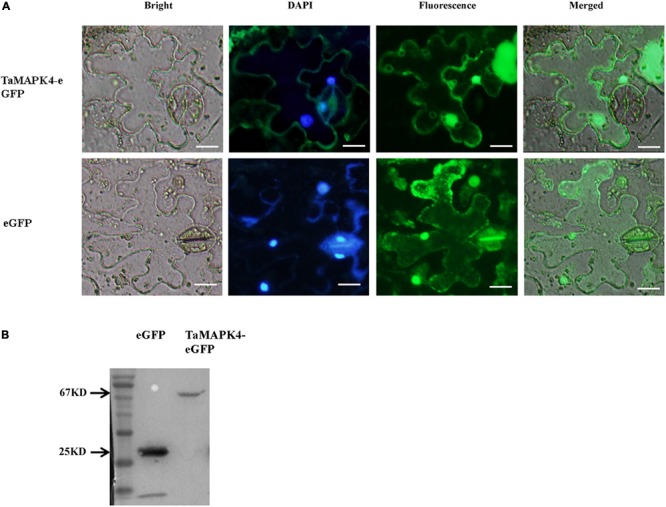
Subcellular localization of TaMAPK4 protein. **(A)** TaMAPK4-GFP fusion protein and green fluorescent protein (GFP) (control) were transiently expressed in *N. benthamiana*. **(B)** Western blot analysis of GFP and TaMAPK4-GFP fusion protein. Bar = 20 μm. Similar results were obtained from three biological replicates.

Prediction analysis revealed that *TaMPK4* is localized in the cytoplasm (Hao et al., 2015). We conducted subcellular localization experiments in *N. benthamiana* leaf cells to better characterize the biological function of *TaMAPK4* and determine the subcellular localization of *TaMAPK4*. Interestingly, when *N. benthamiana* leaves were infused with the pCAMBIA-1302-*TaMAPK4* constructs green fluorescent protein (GFP), fluorescence was transiently expressed in both the cytoplasm and the nucleus, and GFP was uniformly distributed throughout the cell in the control (**Figure 2A**). To confirm the subcellular localization results, we used western blotting to analyze the expression and stability of the TaMAPK4 fusion protein and found that both GFP and TaMAPK4 were successfully expressed (**Figure 2B**). Moreover, we expressed the *TaMAPK4*-GFP fusion protein in wheat protoplasts, found that the GFP fusion protein also expressed in both the cytoplasm and the nucleus (**Supplementary Figure S1**). We inferred that TaMAPK4 might have multiple roles in signaling either from the nucleus or its localization in the cytoplasm.

### Transcriptional Responses of *TaMAPK4* to *Pst* and Hormone Treatment

**FIGURE 3:**
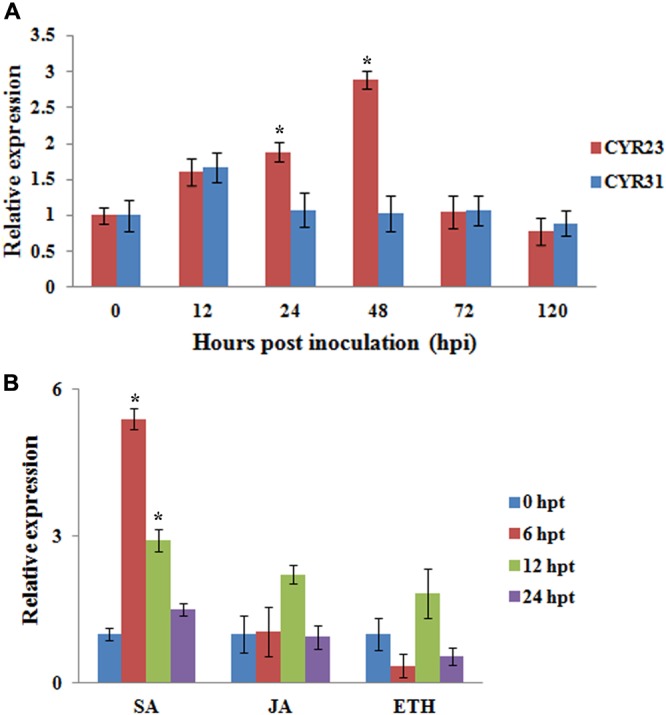
qRT-PCR analysis of relative transcript levels of *TaMAPK4*. **(A)** Transcript profiles of *TaMAPK4* in wheat leaves (Su11) inoculated with *Pst* races CYR31 (virulent) and CYR23 (avirulent), respectively. **(B)** *TaMAPK4* transcript profiles in wheat leaves treated with hormone elicitors. SA, salicylic acid; MeJA, methyl jasmonate; ET, ethylene. Mean expression values were calculated from three independent replicates. Significant differences were determined using Student’s *t*-test: ^∗^*P* < 0.05.

We examined the transcript levels of *TaMAPK4* in *Pst*-infected wheat leaves using quantitative real-time PCR (qRT-PCR). *TaMAPK4* transcript levels increased as early as 12 hpi and exhibited an approximately 2.8-fold peak response at 48 hpi in leaves inoculated with avirulent *Pst* CYR23 (**Figure 3A**). The transcript level of *TaMAPK4* was only up-regulated at 12 hpi, and then showed no obvious change in leaves inoculated with virulent *Pst* CYR31. Therefore, the relative transcript level of *TaMAPK4* in leaves inoculated with CYR23 was higher than in leaves inoculated with CYR31.

We also tested the expression of *TaMAPK4* under methyl jasmonate (MeJA), SA, and ethephon (ET) stresses. *TaMAPK4* transcript levels were strongly elevated, reaching a 5.4-fold peak at 6 hpt in response to SA (**Figure 3B**), but there was no significant change in the transcript levels of *TaMAPK4* following JA treatment. These results suggest that SA signaling was involved in *TaMAPK4* regulation.

### Knockdown of *TaMAPK4*-Decreased Resistance to *Pst*

**FIGURE 4:**
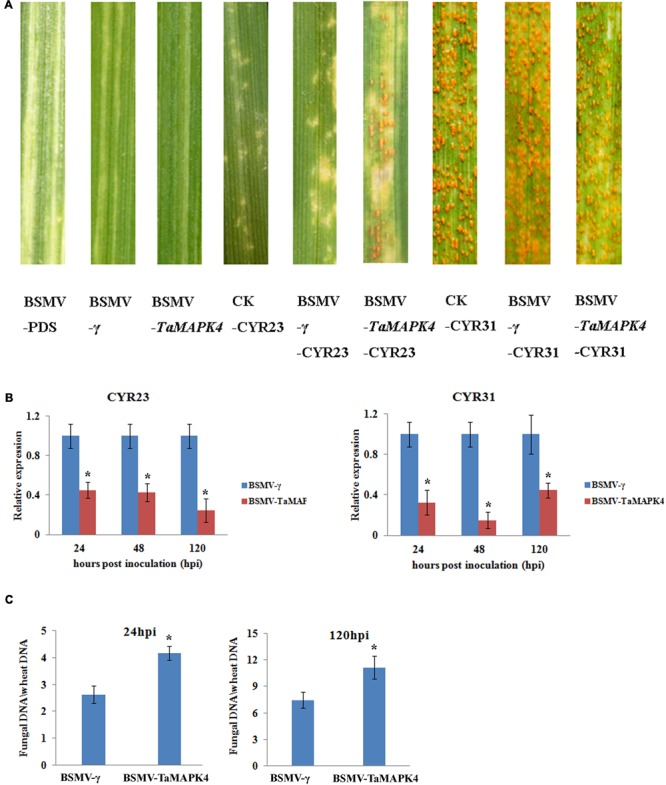
Functional characterization of *TaMAPK4* after virus-induced gene silencing. **(A)** Phenotypic changes in the fourth leaves of plants pre-inoculated with positive control vector (BSMV-PDS), FES buffer (CK), or empty BSMV vector (BSMV-γ) at 14 days post-virus treatment. Phenotypes for the fourth leaves inoculated with *Puccinia striiformis* f. sp. *tritici* (*Pst*) races CYR23, or CYR31 at 14 dpi. **(B)** Relative transcript levels of *TaMAPK4* in *TaMAPK4* knockdown leaves. RNA samples were isolated from *TaMAPK4* knockdown leaves infected with *Pst* races CYR23 (left), or CYR31 (right). BSMV-γ leaves infected with *Pst* races CYR23, or CYR31 were used as control, respectively. **(C)** Biomass of *Pst* (CYR23) measured at 24 and 120 hpi. Means and standard deviations were calculated from three independent replicates. Significant differences were determined using Student’s *t*-test: ^∗^*P* < 0.05.

We used a VIGS system to characterize the function of *TaMAPK4* in the stripe rust-resistant wheat cultivar Su11. The plant displayed mild chlorotic mosaic symptoms and no other obvious defects at 10 days post-virus inoculation (dpi). As a control to confirm that the VIGS system was functioning correctly, we silenced the wheat phytoene desaturase (*PDS*) gene through inoculation with the recombinant virus BSMV: *TaPDS*, causing severe symptoms of chlorophyll photobleaching at 10 dpi. The fourth leaves of Su11 plants were inoculated with avirulent *Pst* CYR23 and virulent CYR31 at 10 dpi with the virus. Conspicuous hypersensitive responses (HR) were elicited by CYR23-infected control seedlings (CK) and seedlings that were previously infected with BSMV: γ. However, there was increased uredinial development and limited urediniospore production in leaves infected with BSMV: *TaMAPK4* at 14 dpi (**Figure 4A**). There was no phenotypic change in the compatible interaction compared with the CK, which exhibited normal disease development and profuse sporulation on all leaves inoculated with CYR31.

qRT-PCR was used to confirm that the RNAi system was functional by determining the silencing efficiency of *TaMAPK4*. Compared to leaves inoculated with BSMV: γ, the transcript levels of *TaMAPK4* were reduced by 55, 58, and 76% at 24, 48, and 120 hpi, respectively, in BSMV: *TaMAPK4* leaves inoculated with CYR23 (**Figure 4B**). In the compatible interaction, the expression of *TaMAPK4* was reduced by 56–86%. The *Pst* biomass was also increased in the leaves of plants with *TaMAPK4* knocked-down (**Figure 4C**). Moreover, we found miR164 was up-regulated in the *TaMAPK4* knocked-down leaves inoculated with CYR23 (**Supplementary Figure S2**).

Su11 leaves inoculated with BSMV: *TaMAPK4* showed increased uredinial development in response to CYR23. To further verify these phenotypes, detailed histological changes were compared to the control (**Table 1**). *Pst* hyphal length and hyphal branches in BSMV: *TaMAPK4* knockdown leaves were obviously longer (*P* < 0.05) than those in the BSMV: γ-treated leaves at 24 and 48 hpi. Moreover, the uredinial area in wheat leaves pre-inoculated with BSMV: *TaMAPK4* was significantly (*P* < 0.05) increased at 120 hpi.

**Table 1:** Histological analysis of *TaMAPK4* knockdown leaves responding to infection by *Pst* race 23.

	Hyphal length (μm)		Hyphal branches		Uredinial area (μm^2^)
Treatment	24 hpi	48 hpi	24 hpi	48 hpi	120 hpi
BSMV-γ	0.24^a^± 0.12	0.33^a^± 0.05	1.34^a^± 0.18	1.65^a^± 0.14	0.21^a^± 0.08
BSMV-*TaMAPK4*	0.34^b^± 0.07	0.44^b^± 0.08	1.53^b^± 0.11	1.93^b^± 0.23	0.29^b^± 0.05

*Leaves were inoculated with BSMV-γ, or BSMV-*TaMAPK4*, followed by inoculation with *Pst* CYR23. hpi, hours post-inoculation.*

*Pst hyphal branches, hyphal length and uredinial area were measured for at least 50 infection sites.*

*Numbers within the same column marked with different superscript letters are significantly different according to one-way ANOVA (*P* < 0.05). Results were obtained in three independent biological replicates.*

### SA Accumulation and Defense-Related Genes in *Tamapk4* Knockdown Wheat Leaves

**FIGURE 5:**
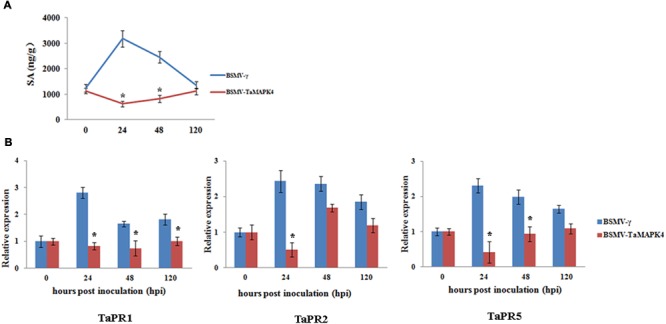
Salicylic acid accumulation and transcript levels of pathogenesis-related (*PR*) genes in *TaMAPK4* knockdown leaves. **(A)** SA accumulation in *TaMAPK4* knockdown leaves inoculated with *Pst* race CYR23. SA, salicylic acid; ng/g, SA accumulation (ng) per fresh leaves weight (g); **(B)** Transcript levels of *TaPR1, TaPR2*, and *TaPR5* genes in *TaMAPK4* knockdown leaves challenged with *Pst* race CYR23. *TaPR1*, pathogenesis-related protein 1; *TaPR2*, β-1,3-glucanase; *TaPR5*, thaumatin-like protein. BSMV-γ leaves infected with *Pst* race CYR23 were used as control. Means and standard deviations were calculated from three independent replicates. Significant differences were determined using Student’s *t*-test: ^∗^*P* < 0.05.

*TaMAPK4* transcript levels were strongly elevated in response to SA. To determine whether the knockdown of *TaMAPK4* affects SA accumulation in wheat leaves, we assessed the SA accumulation in *TaMAPK4* knockdown wheat leaves after infection with *Pst*. SA accumulation was decreased at 24 and 48 hpi (**Figure 5A**).

To determine whether the expression of defense-related genes was affected by *TaMAPK4* silencing, we selected *PR* genes for qRT-PCR analysis. The transcript levels of *TaPR1, TaPR2*, and *TaPR5* were decreased in *TaMAPK4* knockdown plants infected with the *Pst* pathotype CYR23 (**Figure 5B**). These results indicated that *TaMAPK4* might affect the transcript levels of defense-related genes in the wheat-*Pst* interaction.

## Discussion

Mitogen-activated protein kinases are regulated by the sequential phosphorylation of MAPK kinase (Zhang and Klessig, 2001). In the present study, we verified that *TaMAPK4* is a candidate target of miR164 by co-expression studies in *N. benthamiana*. A single miRNA could regulate multiple target genes with different roles in plant immunity. For example, miR863-3p, which targets two atypical receptor-like pseudokinases (ARLPKs) and SERRATE (SE), is induced by the bacterial pathogen *Pseudomonas syringae*. During an early stage of infection, miR863-3p silences the ARLPKs that act as negative regulators of plant defense. During infection, miR863-3p silences SE to positively regulate plant defense (Niu et al., 2016). miR164 also regulates multiple target genes, such as NAC genes and ethylene insensitive 3 (EIN3) (Kim et al., 2009). In a previous study, we observed that *TaNAC21*, a target of miR164, played a negative role in *Pst* resistance in wheat. The expression of *TaNAC21* was induced at 24 and 48 hpi in the compatible interaction (Feng et al., 2014). However, in the present study, the transcript accumulation of *TaMAPK4* was increased at 12, 24, and 48 hpi in the incompatible interaction. Thus, these results suggested that miR164 might regulate target genes in the wheat-*Pst* interaction. *TaMAPK4* and *TaNAC21* played different roles in the regulation of defense in different wheat-*Pst* interactions. However, the detailed mechanisms of the interplay between *TaMAPK4* and miR164 in plant defense need to be further explored.

The characterization of where a gene exerts its function is needed to understand the biological function of the gene. As previously described, there are few reports on the subcellular localization of plant MAPKs (Zaïdi et al., 2010). The mechanism of MAPK localization is complex. It is well-established that MAPKs shuttle between the cytoplasm and nucleus in yeast and mammalian cells in response to different types of stimulation (Cyert, 2001). In plants, Arabidopsis MKP1 was primarily cytoplasmic and interacted with and dephosphorylated MPK6, and MPK1 might be involved in regulating basal MPK activity (Bartels et al., 2009). Moreover, TMPK1 (*Triticum turgidum L. subsp. Durum*) was found in the nucleus, and this protein may travel from the cytoplasm to the nucleus to regulate phosphatase activity and interact with TMPK3 and TMPK6 by associating with NLS-containing proteins, suggesting a regulatory function by interacting with specific nuclear components (Zaïdi et al., 2010). Our localization experiments determined that TaMAPK4 localized to both the cytoplasm and nucleus. Thus, we inferred that TaMAPK4 might have multiple roles in signaling either from the nucleus or its localization in the cytoplasm.

Mitogen-activated protein kinase cascades play central roles in multiple stress responses, including host immunity (Innes, 2001). In the present study, *TaMAPK4* was up-regulated in the incompatible interaction. The knockdown of *TaMAPK4* by VIGS-induced silencing increased uredinial development in the incompatible race CYR23: Su11 response. The histological results were consistent with the *TaMAPK4* gene expression profiles. Taken together, these results indicated that *TaMAPK4* might contribute to the wheat response to *Pst*.

Salicylic acid is an important component of plant defense signaling (Vlot et al., 2009). *BWMK1* (the rice *MAPK* gene) was activated by SA signal and could phosphorylate another transcription factor(s) exclusively associated with SA-mediated gene expression (Cheong et al., 2003). In the present study, we found that *TaMAPK4* was more highly expressed after SA treatment. These data inferred that *TaMAPK4* might be activated by SA. Moreover, MAPK cascades have been implicated in both the regulation of defense hormone biosynthesis and signaling events downstream of hormone sensing. Arabidopsis MPK3 and, to a lesser extent, MPK6 play pivotal roles in the SA-mediated priming of plants for protection against pathogens (Beckers et al., 2009; Bethke et al., 2012). In contrast, Arabidopsis *mpk4* (not the homolog of *TaMAPK4*) mutant plants exhibited constitutively elevated levels of SA, and increased pathogen resistance (Petersen et al., 2000). Moreover, the constitutive SA responses in the *mpk4* mutant were triggered by the SUMM2 (an R protein)-mediated signaling pathway (Zhang et al., 2012). In another experiment, the SA levels in *TaMAPK4* knockdown seedlings in the incompatible interaction were significantly lower than those in the compatible interaction, suggesting that *TaMAPK4* is a positive regulator of SA signaling. Therefore, these results suggested that *TaMAPK4* plays important roles in SA-mediated defense signaling pathways.

The accumulation of PR proteins in the plant is related to plant resistance responses (Van Loon and Van Strien, 2002). MAPK genes are involved in regulating the expression of *PR* genes. For example, the ectopic overexpression of *BWMK1* in transgenic tobacco plants led to increased resistance to pathogens associated with elevated levels of *PR* gene expression (Cheong et al., 2003). We therefore assessed the expression of *PR* genes in *TaMAPK4* knockdown seedlings and found that transcript levels of *TaPR1, TaPR2*, and *TaPR5* were significantly reduced relative to non-knockdown controls. Based on these results, we concluded that the reduced resistance to *Pst* in *TaMAPK4* knockdown wheat plants might be due to decreased defense responses.

## Conclusion

*TaMAPK4* was induced during the wheat-*Pst* incompatible interaction and likely contributed to the actual expression of incompatibility between the wheat host and *Pst* pathogen, referred to as the infection type. Our results suggest that *TaMAPK4* is regulated by miR164, and these data provide further insights into how MAPK genes might be involved in plant defense.

## Author Contributions

BW carried out most of the experiments; BW and ZK wrote the manuscript; NS performed the quantitative RT-PCR and analyzed the data; QZ and NW grew the plant samples; NW collected all the phenotypic data; and ZK revised the manuscript. All authors read and approved the final manuscript.

## Conflict of Interest Statement

The authors declare that the research was conducted in the absence of any commercial or financial relationships that could be construed as a potential conflict of interest.

## Footnotes

1 http://plantgrn.noble.org/psRNATarget/

2 http://www.ibm.com, version 16.0

## Supplementary Material

FIGURE S1 Subcellular localization of the TaMAPK4 protein. GFP and TaMAPK4-GFP fusion proteins were expressed in wheat protoplasts. Bar = 20 μm.

FIGURE S2 Relative transcript levels of miR164 in *TaMAPK4* knockdown leaves infected with *Pst* race CYR23. BSMV-γ leaves infected with *Pst* race CYR23 were used as control. Means and standard deviations were from three independent replicates. Significant differences were determined using Student’s *t*-test: ^∗^*P* < 0.05.

TABLE S1 Prediction of tae-miR164 target in wheat.

TABLE S2 Primers designed for *TaMAPK4* research.
