# Supplementary figures and images for: TaMAPK4 Acts as a Positive Regulator in Defense of Wheat Stripe-Rust Infection
Source: Front Plant Sci. 2018 Feb 15;9:152. doi: 10.3389/fpls.2018.00152 (PMC5829626; doi:10.3389/fpls.2018.00152)

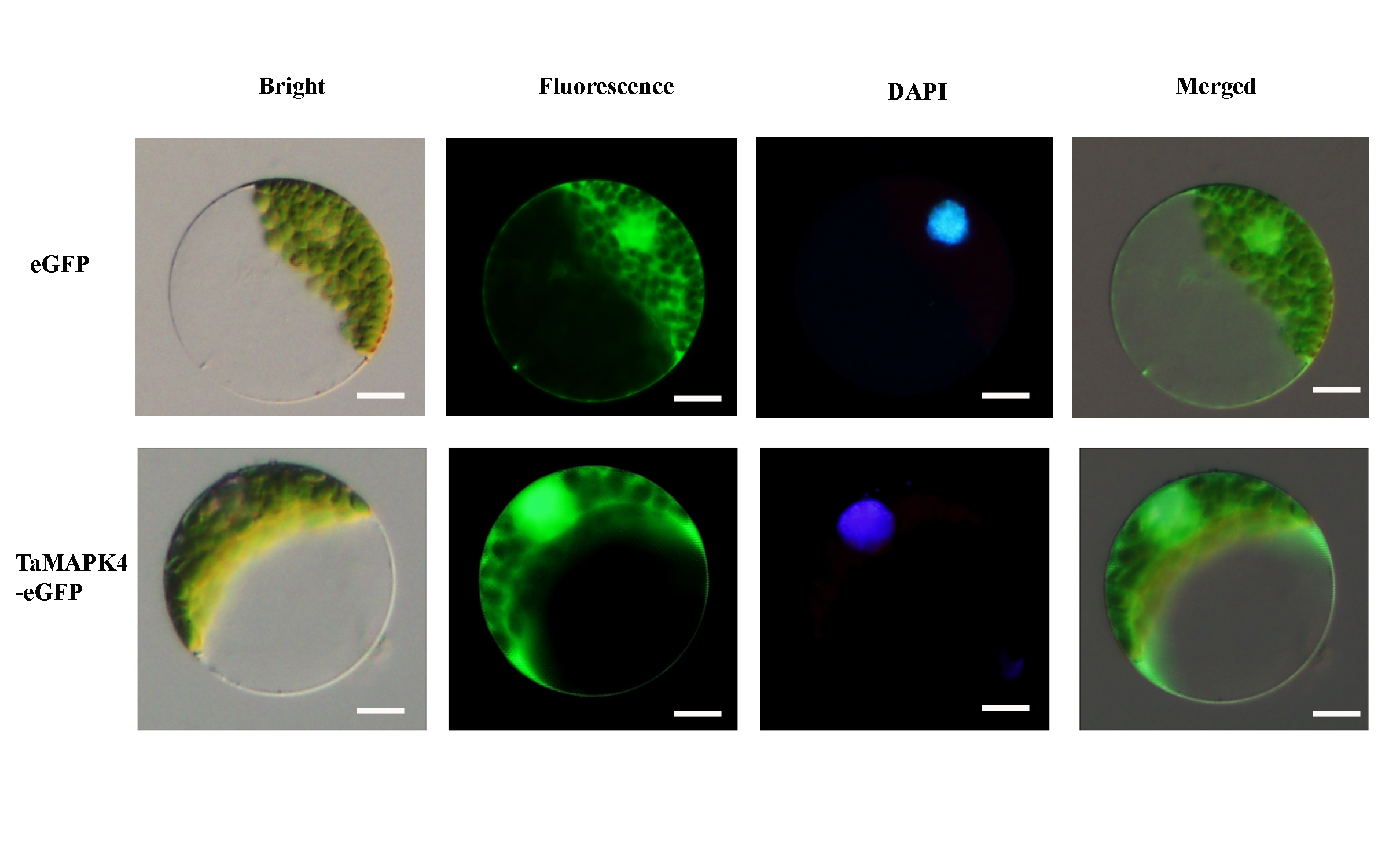

Supplement: FIGURE S1 — Subcellular localization of the TaMAPK4 protein. GFP and TaMAPK4-GFP fusion proteins were expressed in wheat protoplasts. Bar = 20 μm. [file Image_1.TIF]

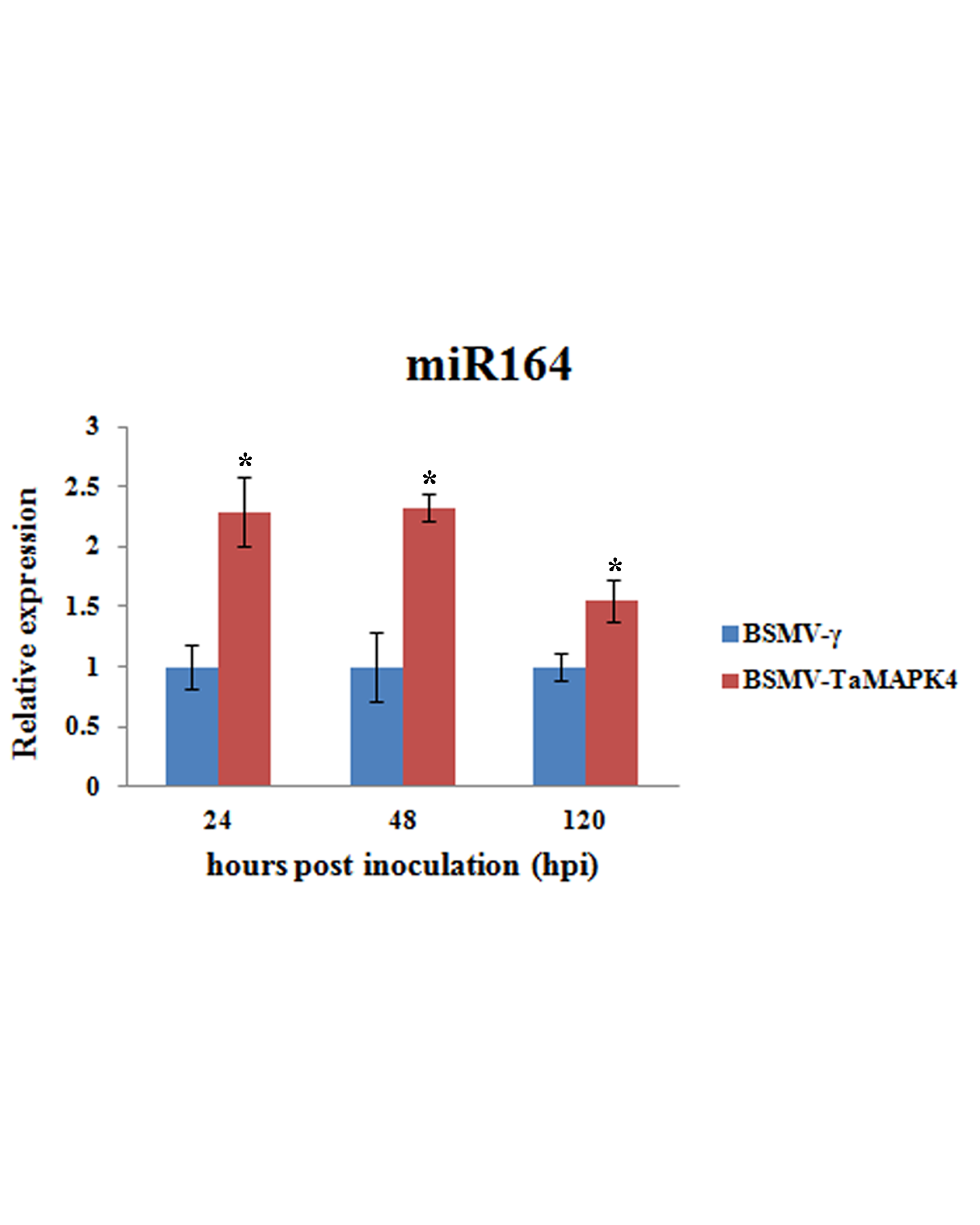

Supplement: FIGURE S2 — Relative transcript levels of miR164 in TaMAPK4 knockdown leaves infected with Pst race CYR23. BSMV-γ leaves infected with Pst race CYR23 were used as control. Means and standard deviations were from three independent replicates. Significant differences were determined using Student’s t-test: ∗P < 0.05. [file Image_2.TIF]
