# Supplementary material for: TaMAPK4 Acts as a Positive Regulator in Defense of Wheat Stripe-Rust Infection
Source: Front Plant Sci. 2018 Feb 15;9:152. doi: 10.3389/fpls.2018.00152 (PMC5829626; doi:10.3389/fpls.2018.00152)
Supplement: TABLE S1 — Prediction of tae-miR164 target in wheat. [file Table_1.DOCX]

| Targets | Score | Alignment | Inhibition |
| --- | --- | --- | --- |
| *TaMAPK4* | 2.5 | ACGUGCACGGGACGAAGAGGU | cleavage |
|  |  | : : : : : : : . : : : : : : : : : : |  |
|  |  | CGGACGUGCUCUGCUUCUCCA |  |
|  |  |  |  |
