# Supplementary material for: TaMAPK4 Acts as a Positive Regulator in Defense of Wheat Stripe-Rust Infection
Source: Front Plant Sci. 2018 Feb 15;9:152. doi: 10.3389/fpls.2018.00152 (PMC5829626; doi:10.3389/fpls.2018.00152)
Supplement: TABLE S2 — Primers designed for TaMAPK4 research. [file Table_2.DOCX]

| ID | Primers (5′-3′) |
| --- | --- |
| TaMAPK4- pCAMBIA-1302-F | AACTGCAGATGGCGATGATGGTGGAT |
| TaMAPK4- pCAMBIA-1302-R | GCTCTAGATCACATGTTCATTCCTGCTT |
| TaMAPK4-VIGS-F | ATATTAATTAAGAAACCACGGGAAGCACTAC |
| TaMAPK4-VIGS-R | TATGCGGCCGCTGGCGACCTTCTCGTTGG |
| GZH-164-F | GGATCCCGCGCGAGGTGGAGAAGCAG |
| GZH-164-R | GTCGACCCAAAACACCAAATAACCGCG |
| GZH-TaMAPK4-F | GCTCTAGATCAGGTTCCAGACCGCCA |
| GZH-TaMAPK4-R | CGGGATCCCAGCCTGACGATTCCTTCTT |
| TaMAPK4-qRT-F | GCCAAGAAGGAATCGTCAGG |
| TaMAPK4-qRT-R | CCAAACCCCATTTAGTAGGAGTA |
| TaEF-F | TGGTGTCATCAAGCCTGGTATGGT |
| TaEF-R | ACTCATGGTGCATCTCAACGGACT |
